# Multi-stage bidirectional informed-RRT * plant protection UAV path planning method based on A * algorithm domain guidance

**DOI:** 10.3389/fpls.2025.1650007

**Published:** 2025-08-22

**Authors:** Jian Li, Yuan Gao, Zheng Li, Weijian Zhang, Weilin Yu, Yating Hu, He Liu, Changtian Li

**Affiliations:** ^1^ College of Information Technology, Jilin Agricultural University, Changchun, China; ^2^ Jilin Province Cross-Regional Collaborative Innovation Center for Agricultural Intelligent Equipment, Jilin Agricultural University, Changchun, China; ^3^ College of Engineering and Technology, Jilin Agricultural University, Changchun, Jilin, China; ^4^ Engineering Research Center of Edibleand Medicinal Fungi, Ministry of Education, Jilin Agricultural University Changchun, Changchun, China

**Keywords:** precision agriculture, A*-MSRRT* algorithm, adaptive node allocation, path planning, UAV

## Abstract

Traditional path planning algorithms often face problems such as local optimum traps and low monitoring efficiency in agricultural UAV operations, making it difficult to meet the operational requirements of complex environments in modern precision agriculture. Therefore, there is an urgent need to develop an intelligent path planning algorithm. To address this issue, this study proposes an improved Informed-RRT* path planning algorithm guided by domain-partitioned A* algorithm. The proposed algorithm employs a multi-level decomposition strategy to intelligently divide complex paths into a sequence of key sub-segments, and uses an adaptive node density allocation mechanism to dynamically respond to changes in path complexity. Finally, a dual-layer optimization framework is constructed by combining elliptical heuristic sampling with dynamic weight adjustment. Complex maps are constructed in simulation to evaluate the algorithm’s performance under varying obstacle densities. Experimental results show that, compared to traditional RRT* and its improved variants, the proposed algorithm reduces computation time by 56.3%–92.5% and shortens path length by 0.42%–8.5%, while also demonstrating superior path smoothness and feasibility, as well as a more balanced distribution of search nodes. Comprehensive analysis indicates that the A*-MSRRT* (A*-Guided Multi-stage Bidirectional Informed-RRT*) algorithm has strong potential for application in complex agricultural environments.

## 1 Introduction

With the rapid advancement of UAV technology, its applications in the field of precision agriculture are becoming increasingly widespread (Guebsi et al., 2024), particularly demonstrating strong potential in crop growth assessment (Fan et al., 2022), yield prediction (Fang et al., 2024; van Klompenburg et al., 2020), and pest and disease early warning (Yang, 2020). Utilizing various types of sensors, UAVs can quickly acquire key physiological and biological parameters of crops, significantly enhancing data collection efficiency (Rejeb et al., 2022). The acquired information provides a solid data foundation for agricultural production, gradually making UAVs a core tool in promoting intelligent and smart and precise agricultural management (Tsouros et al., 2019).

However, in most agricultural operation environments, UAVs often encounter interference from complex obstacles such as dense trees during ultra-low altitude flight missions, which severely restricts their operational efficiency and flight safety (Aliloo et al., 2024). To overcome these challenges, many researchers have carried out comprehensive investigations into various path planning strategies (Yang et al., 2023), aiming to enhance UAVs’ autonomous navigation and obstacle avoidance capabilities in high-density obstacle environments (Fang et al., 2021; Yedilkhan et al., 2024). Representative methods include heuristic search algorithms such as A* (Foead et al., 2021; Hart et al., 1968), graph search-based algorithms like Dijkstra (Dijkstra, 1959; Peyer et al., 2009), and sampling-based algorithms exemplified by Rapidly-exploring Random Tree (RRT) (Varricchio et al., 2014). Owing to the high complexity and dynamic nature of agricultural environments, the A* algorithm encounters significant challenges in designing effective heuristic functions and suffers from exponential increases in computational complexity when operating within high-dimensional configuration spaces (Henkel et al., 2016). The Dijkstra algorithm ensures an optimal solution by exhaustively traversing the entire graph space. In comparison, the RRT algorithm, with its straightforward structure and high computational efficiency, is capable of quickly generating feasible paths in high-dimensional spaces, making it well-suited for path planning in complex scenarios. Consequently, RRT has progressively emerged as a representative approach in intelligent path planning research and holds a prominent position among various planning algorithms (Wu et al., 2021). Due to its strong pathfinding capability in complex environments, this study adopts the RRT algorithm as the foundational framework for further enhancement, aiming to improve its practical effectiveness in agricultural UAV applications.

Nevertheless, the RRT* algorithm requires the exploration of numerous nodes, resulting in higher computational costs and reduced efficiency in the path planning process. As the map size expands, the computational load increases exponentially. To tackle this issue, extensive studies have been carried out by researchers worldwide to optimize and improve such algorithms. YanLin et al. proposed HBAI-RRT*, a dual-tree search method with greedy heuristics that speeds up convergence but is complex and heavily heuristic-dependent (Lin and Zhang, 2024). Tai Huang et al. proposed PF-RRT*, combining APF and RRT* to improve path efficiency and quality, though its performance depends heavily on specific environments (Huang et al., 2023). Peng Xin proposed an enhanced bidirectional RRT* algorithm integrating APF and DWA to improve path quality and planning efficiency, though its performance remains limited in highly complex environments (Xin et al., 2023). Xinyan Chen et al. proposed an Informed-RRT* algorithm that uses sub-nodes as intermediate points in combination with a dual-directional search strategy (Chen et al., 2025), which reduces computation time and path length and improves efficiency. However, its application scenarios are relatively simple and not appropriate for path planning in environments with high obstacle density.

Although current enhanced RRT* algorithms have achieved certain improvements in planning efficiency and path quality, they perform poorly in high-obstacle-density environments. These methods often suffer from overly complex structures, rigid reliance on predefined sampling regions, and limited adaptability, resulting in frequent path failures, inefficient exploration, and suboptimal trajectory structures lacking robustness and continuity (Sánchez-Ibáñez et al., 2021). As a result, it remains difficult to balance planning efficiency and path quality. Therefore, this study aims to enhance the efficiency and adaptability of the RRT* algorithm in complex agricultural environments by integrating an A*-based key node selection mechanism with an optimized multi-stage path decomposition strategy, effectively addressing the poor performance of existing methods in high-obstacle-density scenarios.

The main contributions of this study are as follows:

1. In order to improve the low global search efficiency of conventional RRT algorithms in complex scenarios, this study proposes a heuristic-based key node extraction method. By employing the A* algorithm to quickly identify a near-optimal path, several key intermediate nodes along the path are selected as key intermediate nodes for segmenting the trajectory. In this way, the path planning problem is divided into multiple relatively independent and spatially localized sub-tasks, significantly reducing the dimensionality and complexity of the global search space.
2. To address the issue of uneven distribution of computational resources in multi-stage path planning, this study proposes an adaptive node allocation method for corner optimization. The algorithm establishes a node prioritization model based on turning angles to accurately identify key turning points, and ensures uniform node distribution through adaptive distance constraints and a path length balancing algorithm. Additionally, a dynamic iterative resource allocation strategy is constructed to allocate computational resources rationally according to the complexity weights of each path segment.
3. This study further proposes a segmented bidirectional Informed-RRT* path optimization algorithm. For each pair of adjacent key intermediate nodes, a local path segment is independently constructed by initializing bidirectional search trees and elliptical samplers, thereby enabling efficient path optimization within each local region. Finally, multiple path segments are sequentially stitched together to generate a globally high-quality and feasible path, meeting the dynamic path planning requirements in complex obstacle-dense and multi-channel environments.

## 2 Materials and methods

### 2.1 Problem definition

The path planning problem is defined as follows: Let the configuration space be $X\;=\;R^{d}$ , which is an important concept in UAV path planning, where the obstacle region is denoted as $X_{obs}\subset X$ , and the free space as $X_{free}=X\setminus X_{obs}$ . The distance between nodes $x_{1},\;x_{2}\in X$ is measured using the standard Euclidean norm $x_{1}-x_{2}$ . Let $\;x_{star}\;\in \;X_{free}$ be the starting position and $x_{goal}\in X_{free}$ be the target position, then the path planning problem can be formalized as a triplet $\left(X_{free},x_{star},x_{goal}\right)$ . The path is represented as a continuous mapping $\beta :[0,1]\to X_{free}$ , satisfying $\beta (0)=x_{start}$ , $\beta (1)=\;x_{goal}$ .

The path planning problem can be divided into the following three sub-problems:

Problem A: Given the triplet $\left(X_{free},x_{star},x_{goal}\right)$ , find a path β:[0,1]→X that connects the start and end points;

Problem B: Ensure that the generated path β satisfies the collision-free constraint throughout the entire path segment T∈[[0,1] , i.e., $\beta (\tau )\in \;X_{tree}$ ;

Problem C: Under the premise of ensuring path feasibility, find a path *t* that satisfies the above conditions in the shortest possible time.

### 2.2 RRT* algorithm

The traditional RRT algorithm often fails to generate globally shortest or optimal paths. To address this, Karaman et al. proposed the classical RRT* algorithm in 2011, which is an improvement over the traditional RRT algorithm (Fang et al., 2023, Fang et al., 2020; Karaman and Frazzoli, 2011; Xu et al., 2025). After a new node is generated, the algorithm does not directly enter the next iteration; instead, it performs collision checking to reselect the most suitable parent node and replans the path accordingly. As illustrated in Appendix S1 (**Supplementary Figure 1**), the black circular markers denote individual nodes, with the labels indicating their respective generation sequence.

The enhanced performance of the RRT* algorithm can be illustrated by assigning weights to a directed weighted graph. The novel approach of resetting and reselecting the parent node during the rewiring process is depicted in Appendix S1 (**Supplementary Figure 1**). The algorithm resets the parent node by finding a path from the start point to the new node with the minimum cost. $x_{rand}$ random point $x_{rand}$ is generated, and the nearest node to $x_{rand}$ on the tree is identified as $x_{nearest}$ . Then, $x_{rand}$ and $\;x_{nearest}$ are connected, and with $x_{rand}$ as the center and *r* as the radius, neighboring nodes are searched on the tree. $x_{rand}$ set of potential parent nodes is identified with the aim of updating $x_{rand}$ and checking for better parent nodes. Starting from a potential parent node $x_{near}$ , the path cost is calculated separately for $x_{nearest}$ and $x_{near}$ as parent nodes. If the new path has a lower cost, the previous edge in the tree is removed, as shown by the dashed line in the figure. The new cost C" is calculated using the following expression when the neighboring node is $x_{near}$


(1) $$\begin{array}{c} C^{\prime }=Cost(x_{near})+Line(x_{near}+x_{new}) \end{array}$$

As shown in Equation 1, where:

$Cost(x_{near})$ is the accumulated cost from the root of the tree to point $x_{near}$ , and $Line(x_{near}+x_{new})$ is the distance from $x_{near}$ to $x_{new}$ . Updating the parent node: if $C"<Cost(x_{new})$ , then update the cost and parent node of $x_{new}$ as shown in Equation 2


(2) $$\begin{array}{c} Cost(x_{new})=C^{\prime }Parent(x_{new})=x_{near} \end{array}$$

If a more optimal parent node is identified, a connection is then established between them. For rewiring, for each neighboring node $X_{near}$ , the cost through the new node $x_{new}$ is calculated as shown in Equation 3


(3) $$\begin{array}{c} C^{\prime }=Cost(x_{new})+Line(x_{new},x_{near}) \end{array}$$

If $C"<Cost(x_{near})$ , then rewiring is performed, and the original connection is removed.

### 2.3 Bidirectional RRT* algorithm

Due to the RRT* algorithm expands the search tree in only one direction and converges relatively slowly, the Bidirectional Rapidly-exploring Random Tree (Bidirectional RRT*) algorithm was introduced as an improved variant to overcome these limitations (Candemir et al., 2024; Liu et al., 2019), aimed at accelerating convergence in high-dimensional configuration spaces. As shown in Appendix S1 (**Supplementary Figure 2**), the algorithm simultaneously builds two exploration trees, T1 from the start point $x_{start}$ and T2 from the goal point $x_{goal}$ , and explores the space through bidirectional expansion. During every iteration, the algorithm randomly selects a point $x_{rand}$ within the configuration space, locates the nearest node $x_{nearest}$ in both trees, and generates a new node $x_{new}$ in its direction. Tree T1 expands outward from $x_{start}$ , while tree T2 grows in reverse from $x_{goal}$ . When the newly generated nodes from both trees are close enough and the path between them is free of collisions, the algorithm proceeds to connect the two trees. Unlike the conventional RRT method, Bidirectional RRT* incorporates a path refinement strategy during tree expansion. Upon generating a new node, it examines neighboring nodes within a defined radius to identify several potential parent candidates, then connects to the one offering the lowest cost, thereby enhancing path quality, as described in Equation 4.


(4) $$\begin{array}{c} Cost(x_{new})=\underset{x_{i}\in N(x_{new})}{\min}[Cost(x_{i})+∥x_{i}-x_{new}{∥}_{2}] \end{array}$$

Each time a new node $x_{new}$ is generated, the algorithm searches its neighboring node set $N(x_{new})$ to find the optimal parent node and connects it through the path with the lowest total cost, as shown in Equation 5


(5) $$\begin{array}{c} P_{total}=P_{T_{1}}(x_{new})\cup P_{T_{2}}(x_{new}^{＇}) \end{array}$$

Once the new nodes $x_{new}$ and $x_{new}^{＇}$ in the two trees are successfully connected, the algorithm traces back from each of these nodes to their respective root nodes (start or goal), resulting in two sub-paths $P_{T_{1}}$ and $P_{T_{2}}$ . The final path is formed by concatenating these two paths into $P_{total}$ .

### 2.4 Informed-RRT* algorithm

Due to the built-in constraints of the RRT* algorithm, including its high computational complexity and reduced search efficiency in high-dimensional environments, Informed-RRT* was proposed by Jonathan D. Gammell and S. Srinivasa (Gammell et al., 2014). It improves the original RRT* algorithm by optimizing the search process for asymptotically optimal paths. When solving path planning problems (Fang and Xie, 2024; Huang et al., 2025; Qureshi and Ayaz, 2015), Informed-RRT* demonstrates faster convergence toward the optimal solution, as illustrated in Appendix S1 (**Supplementary Figure 3**).The traditional Informed-RRT* algorithm utilizes known information (such as the currently known best path length) to constrain the search space, thereby avoiding unnecessary exploration. After finding an initial path, Informed-RRT* uses the current path length $c_{best}$ , the start point $x_{start}$ , and the shortest distance $c_{min}$ between the goal point $x_{goal}$ and the start point. The major axis length is *a*, and the minor axis length is *b*. Let a be equal to half the length of the initial path, as shown in Equation 6


(6) $$\left\{ \begin{array}{c} \begin{array}{c} a=\frac{C_{best}}{2}\\ c=\frac{C_{min}}{2}\\ b=\frac{\sqrt{C_{best}^{2}-C_{min}^{2}}}{2} \end{array} \end{array}\right.$$

An elliptical sampling region is thus constructed. In subsequent iterations, each time a shorter path is found, its length is used as the new $c_{best}$ to update the sampling ellipse, as shown in Appendix S1 (**Supplementary Figure 4**).

### 2.5 A* heuristic key path node extraction strategy

**Figure 1:**
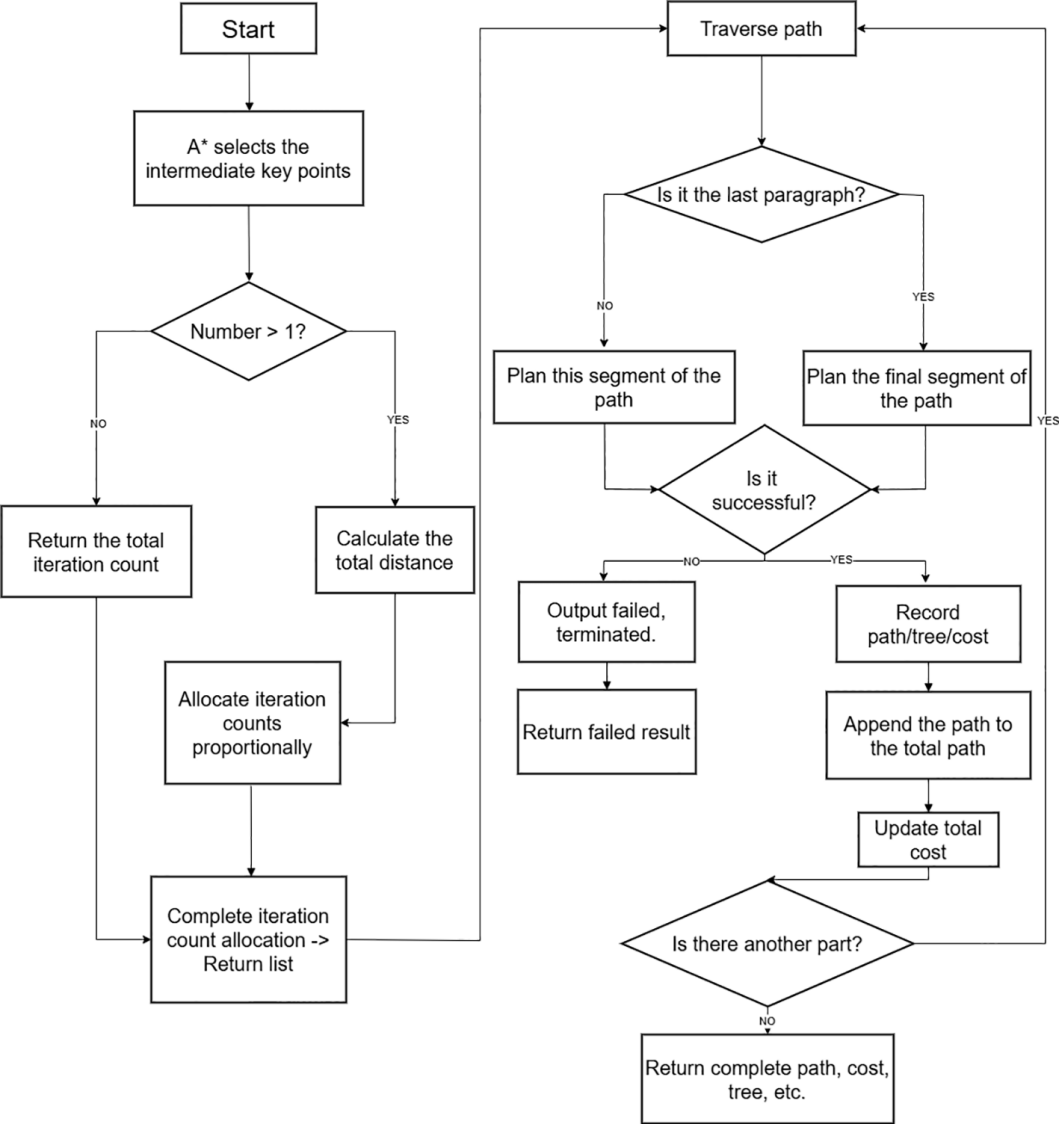
Intermediate sampling point process.

As a core factor determining the convergence performance and route quality of the RRT* algorithm, the node selection strategy suffers from limitations in random sampling efficiency, avoidance of local optima, and adaptability to high-dimensional spaces, which have become key bottlenecks restricting algorithmic performance improvements. This study proposes an optimized intermediate node selection algorithm, aiming to use a dynamically scored mechanism based on corner angles to accurately identify key intermediate nodes in the path. A grid distance constraint is introduced to prevent key intermediate nodes from being overly dense. A new path segment length balancing mechanism is added to automatically supplement necessary key intermediate nodes. Then, by combining A* grid paths with continuous space validation, the validity of key intermediate nodes is ensured. Finally, a visibility check is used to further optimize the number of nodes by removing redundant points. The corresponding pseudocode for this process is presented in Key Waypoints Selection Algorithm, while the detailed procedural steps are visualized in **Figure 1**.

Key waypoints selection Algorithm 1:

Algorithm 1 **Key waypoints selection.**

**Input:** *start, goal*, *obstacle*, *map_info*

**Output:** *key_points*
1: *path* ← *Generate A* ∗ *grid path* (*start*, *goal*, *binary_map*)
2: *simplifiedPath* ← *Remove redundant turning points* (*path*)
3: *scores* ← *Compute node importance* (*simplifiedPath*)
4: *keyPoints* ← [*start*, *goal*]
5: *for each node in sorted* (*simplifiedPath*, *scores descending*) *do*
6:    *
**if** scores* [*node*] > 015 ∧ *min_grid_distance* (*node*, *keyPoints*) ≥ 3 *
**then**
*
7:     *keyPoints.append* (*node*)
8: *
**end**
*
9: *i* ← 0
10: *
**while** i* < *lenght* (*keyPoints*) − 2 **do**
11: ***if** IsVisible* (*keyPoints*[*i*], *keyPoints*[*i* + 2] , *binary_map*) *
**then**
*
12:     *Remove keyPoints*[*i* + 1]
13:    *
**else** i* ← *i* + 1
14: ***end**
*
15: *keyPoints* ← *Insert intermidiate nodes* (*keyPoints*, *original_path*, *max_lenght* = 8.0)
16: ***return** ConvertToContinuousSpace* (*keyPoints*, *obstacles*, *grid_params*)

The A* algorithm is a heuristic-based search method that merges the optimality assurance of the Dijkstra algorithm with the high efficiency of greedy best-first search. It steers the search direction using an evaluation function composed of the actual cost and an estimated heuristic cost. The essence of the A* algorithm is reflected in this evaluation mechanism, which drives the search process, as illustrated in Equation 7


(7) $$\begin{array}{c} f(n)=g(n)+h(n) \end{array}$$

Where: f(n) denotes the total estimated cost of node n; g(n) denotes the actual path cost from the start point to node n; H denotes the heuristic estimated cost from node n to the goal point.

To ensure the effectiveness and accuracy of the algorithm in a two-dimensional grid environment, Euclidean distance is adopted as the heuristic function, as shown in Equation 8


(8) $$\begin{array}{c} h(n)=\sqrt{{\left(x_{n}-x_{goal}\right)}^{2}+{\left(y_{n}-y_{goal}\right)}^{2}} \end{array}$$

Where $\left(x_{n},y_{n}\right)$ and $\left(y_{goal},x_{goal}\right)$ represent the coordinates of the current node and the goal node, respectively. This heuristic function satisfies the admissibility condition, i.e., $h(n)\leq h^{*}(n)$ , where $h^{*}(n)$ is the true optimal cost from node *n* to the goal.

In the actual path search process, it is necessary to continuously update the accumulated cost from the start point to the current node. For grid environments, the update rule of this accumulated cost is given in Equation 9


(9) $$\begin{array}{c} g(n_{neighbor})=g(n_{current})+g(n_{current},n_{neighbor}) \end{array}$$

To extract key nodes from the original path obtained by A* search, a path simplification method based on direction change detection is used. Let the original path be $P=\{P_{0},P_{1},P_{2}\cdots ,P_{m}\}$ , where $p_{i}=(x_{i},y_{i})$ denotes the *i*-th node on the path. The direction vector of adjacent path segments is defined as shown in Equation 10


(10) $$\begin{array}{c} \vec{d_{i}}=(x_{i+1}-x_{i},y_{i+1}-y_{i}) \end{array}$$

When a change occurs between adjacent direction vectors, i.e., ${\vec{d}}_{i-1}\neq {\vec{d}}_{i}$ , the node $p_{i}$ is identified as a critical turning point and retained in the simplified path.

Since the A* algorithm operates in discrete grid space, while actual path planning applications often require continuous space, coordinate system conversion becomes a necessary step. The conversion from grid coordinates  (i, j)  to continuous world coordinates  (x, y)  needs to consider the grid resolution and the geometric characteristics of the map. The conversion formulas are shown in Equations 11 and Equations 12


(11) $$\begin{array}{c} x=(i+0.5)\times resolution \end{array}$$

(12) $$\begin{array}{c} y=(grid_{h}eigh-\text{j}-0.5)\times resolution \end{array}$$

Where resolution denotes the grid resolution, and grid_heigh denotes the height of the grid map.

Based on the above path simplification and coordinate transformation process, a complete mathematical framework for key node extraction can be established. Let the simplified path obtained by the A* algorithm be

$P_{simplified}=\{P_{0},P_{1},P_{2}\cdots ,P_{k}\}$ , where $P_{0}$ and $P_{k}$ represent the start and end points, respectively.

The key intermediate node set is defined as shown in Equation 13.


(13) $$\begin{array}{c} N_{key}=\{p_{i}|i\in \{1,2,\cdots ,k-1\},p_{i}\in P_{simplified}\} \end{array}$$

Each sub-problem is solved within a relatively small local space, thereby significantly improving the overall planning efficiency.

### 2.6 Adaptive node allocation mechanism oriented toward corner optimization

Although the aforementioned optimized intermediate node selection algorithm has achieved significant improvements in node filtering and redundancy elimination, it still faces issues of uneven computational resource allocation and inaccurate path complexity assessment in multi-stage path planning. To address this, this study further proposes an adaptive node allocation mechanism oriented toward corner optimization. By dynamically evaluating the geometric characteristics of path segments and the distribution of obstacles, this mechanism enables intelligent allocation of computational resources, thereby improving planning efficiency and path quality in complex environments. This mechanism first establishes a node importance quantification model based on corner angles. For any three consecutive nodes on the path: $P_{i-1}(x_{i-1},y_{i-1})$ , $P_{i}(x_{i},y_{i})$ , and $P_{i+1}(x_{i+1},y_{i+1})$ , the corner angle is defined and calculated as shown in Equation 14.


(14) $$\begin{array}{c} \theta_{i}=arccos(\frac{{\vec{v}}_{1}\cdot {\vec{v}}_{2}}{\left|{\vec{v}}_{1}|\cdot |{\vec{v}}_{2}\right|}) \end{array}$$

Forward vector and backward vector are respectively defined as shown in Equation 15.


(15) $$\left\{ \begin{array}{c} {\vec{v}}_{1}=(x_{i}-x_{i-1},y_{i}-y_{i-1})\\ {\vec{v}}_{2}=(x_{i+1}-x_{i},y_{i+1}-y_{i}) \end{array}\right.$$

The magnitude of the vector is calculated as shown in Equation 16.


(16) $$\left\{ \begin{array}{c} |{\vec{v}}_{1}|=\sqrt{{\left(x_{i}-x_{i-1}\right)}^{2}+{\left(y_{i}-y_{i-1}\right)}^{2}}\\ |{\vec{v}}_{2}|=\sqrt{{\left(x_{i+1}-x_{i}\right)}^{2}+{\left(y_{i+1}-y_{i}\right)}^{2}} \end{array}\right.$$

The dot product of the vectors is calculated as shown in Equation 17.


(17) $$\begin{array}{c} {\vec{v}}_{1}\cdot {\vec{v}}_{2}=(x_{i}-x_{i-1})(x_{i+1}-x_{i})+(y_{i}-y_{i-1})(y_{i+1}-y_{i}) \end{array}$$

This equation determines the turning angle at a given path node by applying the cosine law to the angle formed between vectors, thereby accurately measuring the curvature at that location. Using the calculated corner angle, a node importance scoring function is defined, as presented in Equation 18.


(18) $$\begin{array}{c} S_{i}=\frac{\theta_{i}}{\pi }+\alpha \cdot D_{i} \end{array}$$

Where $S_{i}$ denotes the importance score, α is the weighting coefficient for obstacle imagery, and $D_{i}$ denotes the inverse of the normalized distance between the node and its nearest obstacle. This scoring function comprehensively considers the geometric features of the path and environmental constraints, achieving accurate identification of key key intermediate nodes.However, relying solely on the importance score may lead to excessive clustering of key nodes. Therefore, to prevent over-concentration of key nodes, an adaptive constraint mechanism based on grid distance is introduced. The grid distance between two nodes is defined as shown in Equation 19


(19) $$\begin{array}{c} d_{grid}(P_{i},P_{j})=|x_{i}-x_{j}|+|y_{i}-y_{j}| \end{array}$$

During the node filtering process, a dynamic distance threshold is applied, as shown in Equation 20


(20) $$\begin{array}{c} T_{min}=max(T_{base},T_{end}\cdot e^{-\frac{d_{end}}{L_{ref}}}) \end{array}$$

Where $T_{base}$ is the base distance threshold, $T_{end}$ is the goal area threshold, $d_{end}$ is the current distance from the node to the goal, and $L_{ref}$ is the reference length. This formula achieves adaptive adjustment of the distance threshold through an exponential decay function, effectively avoiding redundant node distribution near the goal point.

Considering that node filtering may result in uneven segment lengths, a balancing mechanism based on Euclidean distance is designed. For two adjacent key nodes $P_{k}(x_{k},y_{k})$ and $P_{k+1}(x_{k+1},y_{k+1})$ , the distance between them is calculated as shown in Equation 21.


(21) $$\begin{array}{c} L_{segment}=\sqrt{{\left(x_{k+1}-x_{k}\right)}^{2}+{\left(y_{k+1}-y_{k}\right)}^{2}} \end{array}$$

When $L_{segment}>L_{max}$ , the system automatically inserts key intermediate nodes into the original path. The quantity of inserted nodes is calculated using the following expression, as defined in Equation 22.


(22) $$\begin{array}{c} N_{insert}=\frac{L_{segment}}{L_{max}} \end{array}$$

The index interval for the inserted nodes is given by Equation 23.


(23) $$\begin{array}{c} \Delta_{idx}=max(1,\lfloor \frac{idx_{end}-idx_{start}}{N_{insert+1}}\rfloor ) \end{array}$$

Where $idx_{start}$ and $idx_{end}$ are the indices of the starting and ending nodes in the original path. This algorithm ensures the uniformity of segment lengths and improves the success rate of subsequent RRT expansion.Finally, to achieve optimized allocation of computational resources, a dynamic iterative resource allocation strategy is established based on path segment geometric complexity and obstacle distribution. For the *j*-th path segment, the complexity weight is calculated as shown in Equation 24


(24) $$\begin{array}{c} w_{j}=L_{j}-r_{j} \end{array}$$

Where $L_{j}$ is the path segment length and $r_{j}$ is the obstacle difficulty coefficient.

The number of iterations allocated to the *j*-th path segment follows a weighted proportional distribution principle, as shown in Equation 25


(25) $$\begin{array}{c} I_{j}=max(I_{min},[\frac{w_{j}}{\sum_{k=1}^{N}w_{k}}\cdot I_{max}]) \end{array}$$

The meanings of the parameters are as follows: $I_{j}$ denotes the number of iterations allocated to the *j*-th path segment, $I_{min}$ denotes the minimum guaranteed number of iterations for each path segment, $I_{max}$ denotes the upper limit of the total number of iterations in the system, *N* denotes the total number of path segments, and $\frac{w_{j}}{\sum_{k=1}^{N}w_{k}}$ denotes the sum of complexity weights of all path segments. This formula achieves proportional allocation of computational resources through weight normalization, while the minimum value constraint ensures that each path segment receives a basic level of computational support, effectively balancing the relationship between computational efficiency and planning quality.

### 2.7 Multi-stage bidirectional informed-RRT* algorithm combined with A* algorithm

**Figure 2:**
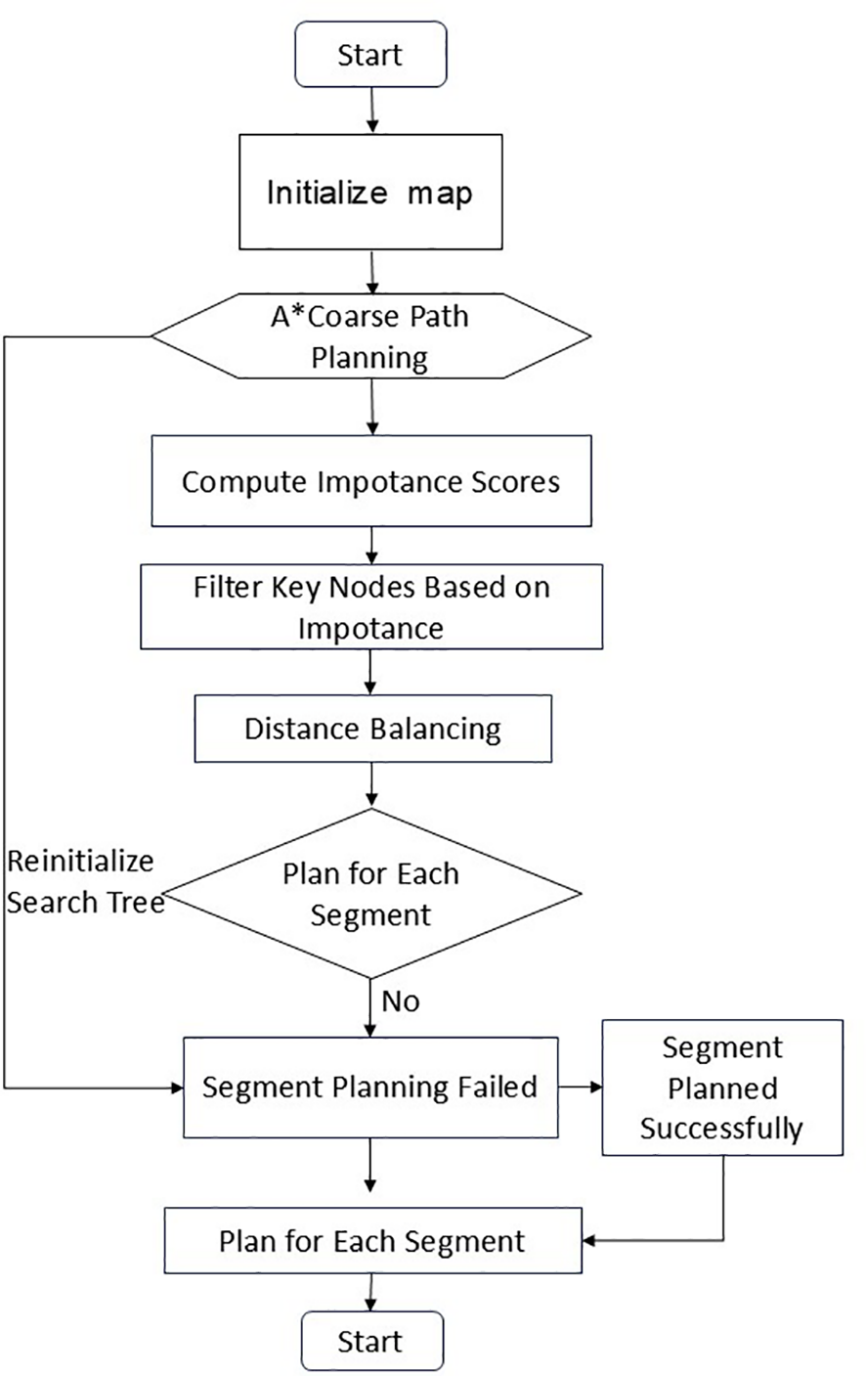
Main procedure of A*-MSRRT* algorithm.

Although the above algorithms have made notable progress in enhancing path planning efficiency and quality, they still face several challenges in agricultural operation scenarios. Specifically, the expansion of the random tree often results in redundant nodes, which diminishes algorithmic efficiency; moreover, the optimization level of the generated paths remains insufficient. To tackle these issues, this research introduces a segmented bidirectional Informed-RRT* algorithm aimed at enhancing the effectiveness of conventional path planning techniques. The primary innovation of the proposed approach is the strategic decomposition of the complex global planning task into multiple locally solvable sub-problems that can be optimized in parallel. By means of mathematical modeling, the initial problem space *X* is partitioned into several sub-domains with clearly defined boundary constraints. In each sub-domain, a dedicated bidirectional search tree is initialized along with an elliptical sampling mechanism. If the local search fails, the corresponding tree is reinitialized and replanned. The complete procedure is illustrated in the flowchart shown in **Figure 2**.

First, under the theoretical framework of the basic RRT algorithm, given the start point $x_{1}\in X_{free}$ and the goal point $x_{2}\in X_{free}$ , the RRT* algorithm constructs a search tree T=(ν,ϵ) , where *v* is the set of nodes and ϵ is the set of edges. For any node a∈ν , the accumulated cost from the root node to this node is defined as shown in Equation 26:


(26) $$\begin{array}{c} c_{a}=\underset{\pi \in \prod (x_{root},a)}{\min}\sum_{i=0}^{|\pi |-1}∥x_{i}-x_{i+1}∥ \end{array}$$

Where $\pi \in \prod (x_{root},a)$ denotes the collection of all valid paths extending from the root node to the given node a .

On this basis, to improve search efficiency, the algorithm adopts a bidirectional search tree construction strategy. This strategy maintains two search trees simultaneously: the forward search tree is $T_{f}=(\nu_{f},\epsilon_{f})$ with the root node $x_{stat}$ , and the backward search tree is $T_{b}=(\nu_{b},\epsilon_{b})$ with the root node as the goal point $x_{goal}$ . The inter-tree connection condition is defined such that for $a_{f}\in \nu_{f}$ and $a_{b}\in \nu_{b}$ , a connection can be established when the following condition is satisfied, as shown in Equation 27:


(27) $$∥\begin{array}{c} a_{f}-a_{b}∥\leq r_{connect}andLine(a_{f},a_{b})\subset X_{free} \end{array}$$

Where $r_{connect}$ is the connection radius threshold, and $\text{L}ine(a_{f},a_{b})$ denotes the straight-line segment connecting the two points.

Furthermore, to address path planning problems in complex environments, the algorithm establishes a segmented path planning mathematical model. Given the start point $x_{stat}$ and the goal point $x_{goal}$ , a key path point sequence is obtained through the intermediate node selection algorithm, as shown in Equation 28:


(28) $$\begin{array}{c} W=\{w_{0},w_{1},\cdots ,w_{k},w_{k+1}\} \end{array}$$

Where $w_{0}=x_{stat}$ , $w_{k+1}=x_{goal}$ , and $w_{i}\in X_{free}$ are intermediate key intermediate nodes. The global path planning problem is thus decomposed into k+1 sub-problems: $P_{i}$ :refers to finding the optimal path from $w_{i}$ to $w_{i+1}$ , i=0,1,⋯,k .

Subsequently, the global path is constructed by connecting the path segments. Let the optimal path of the *i*-th path segment be: $\pi^{\left(i\right)}=\{p_{0}^{\left(i\right)},p_{1}^{\left(i\right)},\cdots ,p_{ni}^{\left(i\right)}\}$ , where $p_{0}^{\left(i\right)}=w_{i}$ and $p_{ni}^{\left(i\right)}=w_{i+1}$ .

The global path is constructed by concatenating the path segments, as shown in Equation 29:


(29) $$\begin{array}{c} \prod global=\pi^{\left(0\right)}⊕\pi^{\left(0\right)}⊕,\cdots ,⊕\pi^{\left(k\right)} \end{array}$$

Finally, the global path is constructed by connecting the path segments. The total cost of the global path is defined as shown in Equation 30:


(30) $$\begin{array}{c} J_{total}=\sum_{i=0}^{k}J^{\left(i\right)} \end{array}$$

Where the cost of the i-th path segment is given by Equation 31:


(31) $$\begin{array}{c} J^{\left(i\right)}=\sum_{i=0}^{k}∥p_{j}^{\left(i\right)}-p_{j+1}^{\left(i\right)}∥ \end{array}$$

This segmented bidirectional Informed-RRT* algorithm decomposes the complex global path planning problem into multiple relatively simple local sub-problems. By applying an efficient elliptical sampling strategy and bidirectional search mechanism within each sub-problem, it achieves rational allocation of computational resources and significantly improves path quality.

## 3 Results

### 3.1 Experimental setup

**Figure 3:**
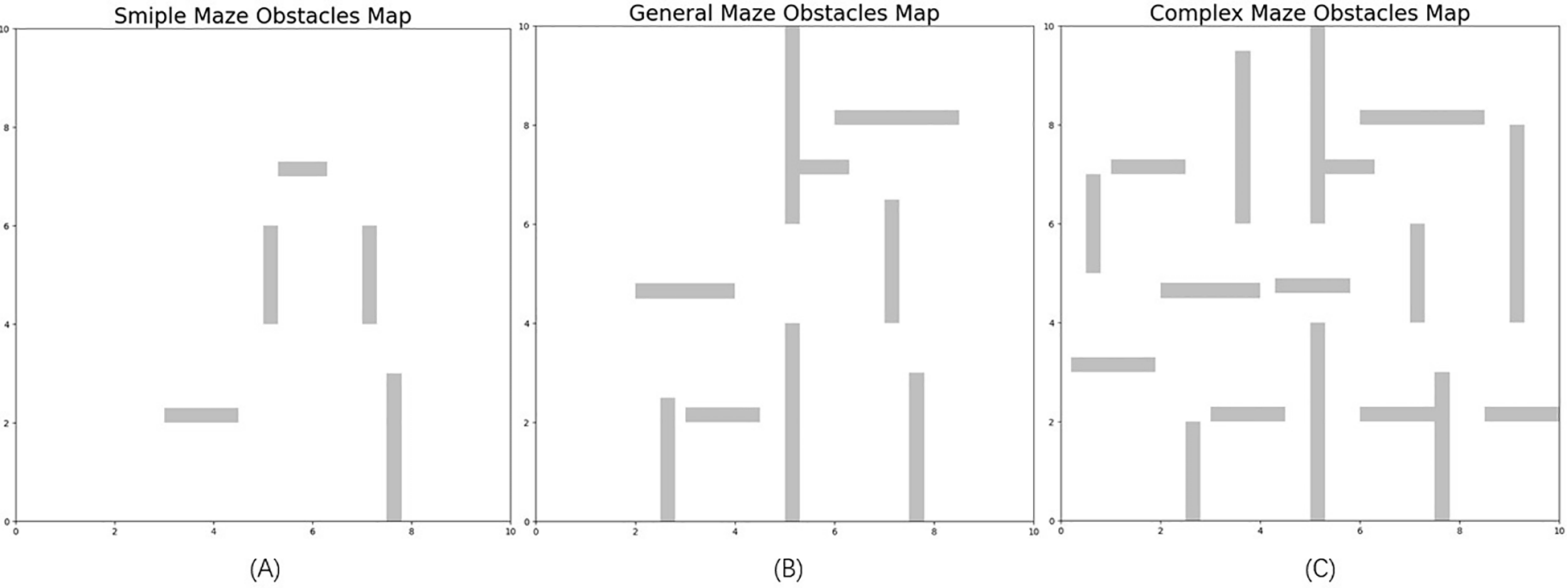
Three different obstacle density environments **(A–C)**.

To evaluate how well the A*-MSRRT* algorithm performs in path planning within environments containing dense obstacles, and to benchmark it against other methods, each algorithm was tested independently 30 times, with the mean values used for comparison across six approaches. The simulation settings encompassed both 2D and 3D environments, sized at 10 × 10 and 90 × 80 × 40, respectively. The expansion step size was set to 0.5. All experiments were conducted using a 2080Ti GPU with Python version 3.8. UAVs were restricted from traversing the light blue obstacle regions in the center. In the bidirectional search process, the blue lines represent the expansion of the tree from the start point, while the purple lines correspond to the growth from the goal point. The updated trajectory after the rewiring phase is indicated by a red dashed line. All other parameters remained unchanged. The proposed method was tested in three distinct scenarios, labeled as Environment A, Environment B, and Environment C, as illustrated in **Figure 3**.

### 3.2 Results and analysis

**Figure 4:**
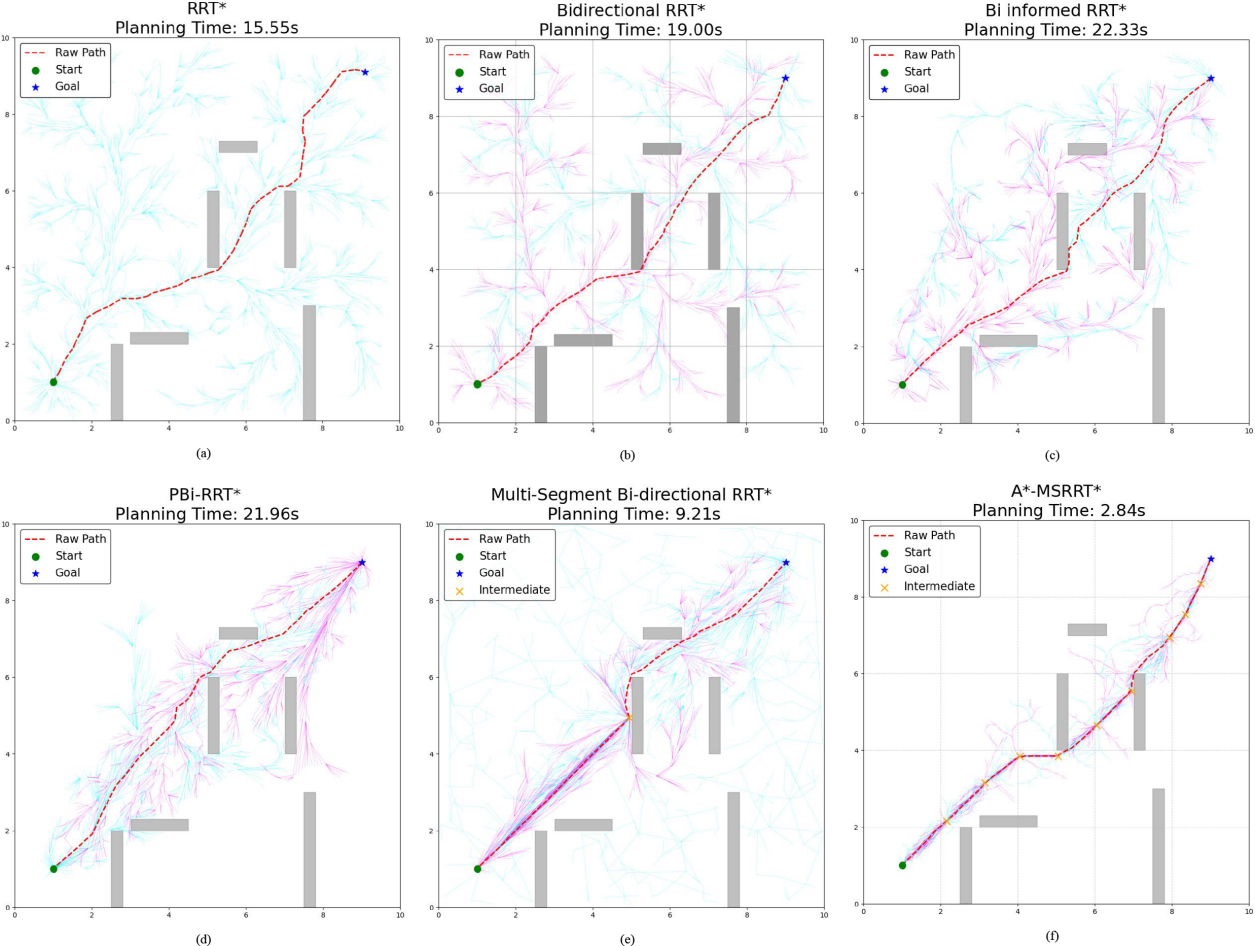
Environment A: low-density obstacles. **(a)** RRT* algorithm, **(b)** Bidirectional RRT* algorithm, **(c)** Bi-Informed-RRT* algorithm, **(d)** PBi-RRT* algorithm, **(e)** MS-Bi RRT* algorithm, and **(f)** A*-MSRRT* algorithm.

This study performed 30 independent trials for each algorithm in complex scenarios to evaluate their stability, recording metrics such as computation time, path length, and node count. The simulation outcomes are presented in **Figure 4**. In **Figure 4a**, while the RRT* algorithm successfully generates a feasible path, the results reveal a lengthy computation time and a trajectory with excessive twists, indicating poor smoothness. **Figure 4b** shows that Bidirectional RRT* results in a shorter path compared to RRT*, but the computation time increases, and path quality remains suboptimal. In **Figure 4c**, Bi-Informed-RRT* requires more time but yields improved path optimization. **Figure 4d** demonstrates that PBi-RRT* produces paths that closely approach obstacles, potentially causing UAV collisions in real-world applications, despite the enhanced path refinement. The MS-Bi RRT* algorithm, as shown in **Figure 4e**, requires fewer nodes, generates shorter paths, and reduces computation time. In contrast, **Figure 4f** illustrates that the A*-MSRRT* algorithm delivers the best overall performance in terms of path length, computational efficiency, and node usage, making it highly suitable for UAV flight tasks. A summary of simulation results in a simplified environment is provided in **Table 1**.

**Table 1:** Comparison of algorithm results in environment A.

Algorithm	Average path length	Average time	Average nodes
RRT*	12.12	15.55	2801
Bidirectional RRT*	11.95	19.00	2781
Bi-Informed-RRT*	12.03	22.33	2617
PBi-RRT*	11.85	21.96	3002
MS-Bi RRT*	11.79	9.21	2511
A*-MSRRT*	11.71	2.84	2214

The asterisk suffix in algorithm names (e.g., RRT, Informed-RRT, A-MSRRT) denotes the asymptotically optimal variant of the base algorithm. This notation originates from the Rapidly-exploring Random Tree Star (RRT)* algorithm proposed by Karaman and Frazzoli (2011), where the asterisk symbolizes its ability to converge to an optimal solution through iterative refinement. In motion planning contexts, the * suffix universally indicates that the algorithm guarantees asymptotic optimality.

According to the tabulated data analysis, the proposed A*-MSRRT* algorithm achieves a trajectory length of 11.71, representing a 3.4% reduction compared to RRT* (12.12), and outperforming all other baseline algorithms in terms of path optimality. In terms of computational efficiency, A*-MSRRT* significantly reduces average computation time to 2.84 seconds, which corresponds to a decrease of 81.7% relative to RRT* (15.55 s), and a reduction ranging from 69.1% (vs. MS-Bi RRT) to 87% (vs. Bi RRT)* compared to other variants. Moreover, the total number of sampled nodes is reduced to 2214, representing an 11.8% to 26.2% decrease compared to all other methods evaluated. These results clearly demonstrate that the proposed algorithm not only minimizes unnecessary random point generation but also achieves substantial improvements in planning efficiency and trajectory quality under complex obstacle-dense environments.

**Figure 5:**
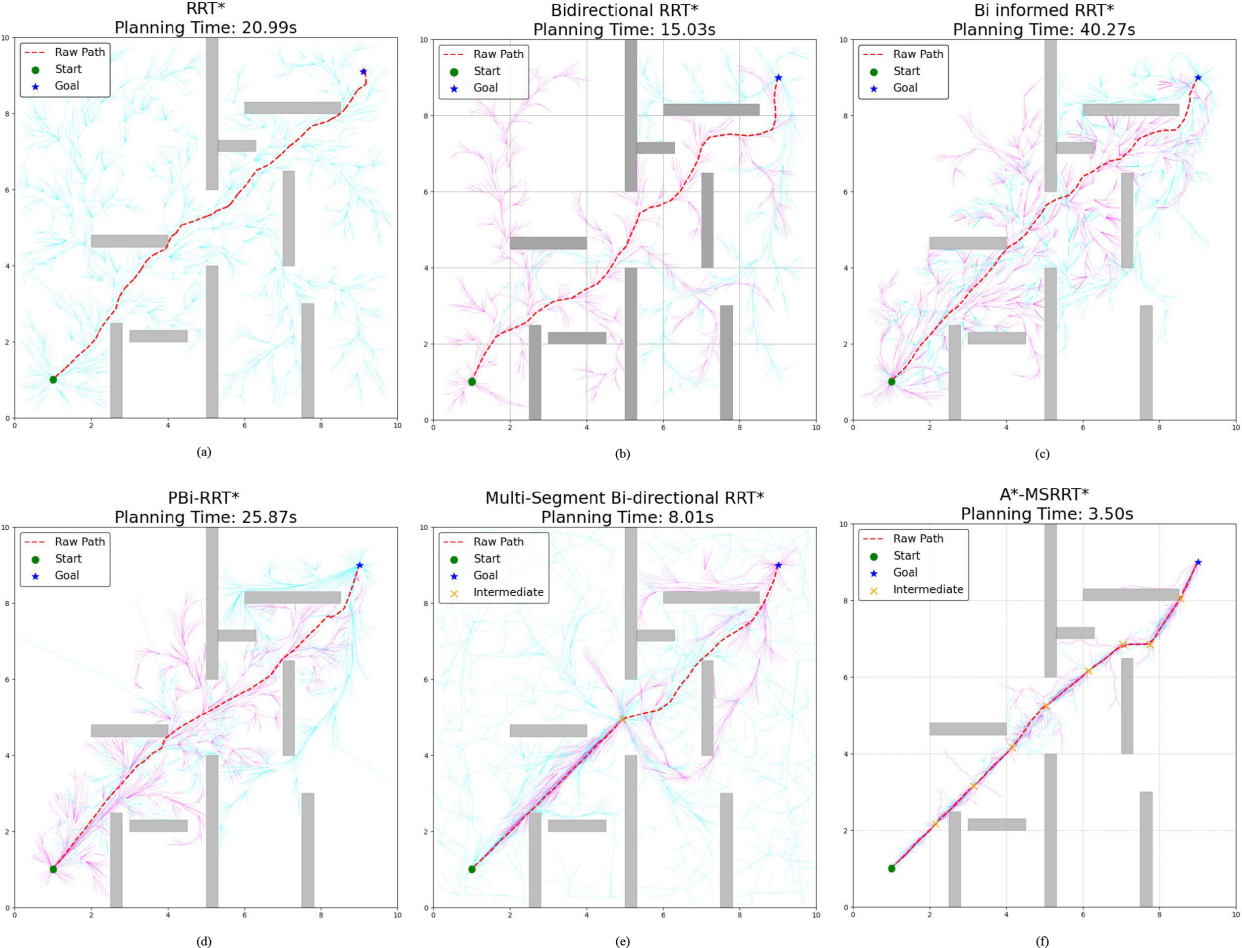
Environment B: medium-density obstacles. **(a)** RRT* algorithm, **(b)** Bidirectional RRT* algorithm, **(c)** Bi-Informed-RRT* algorithm, **(d)** PBi-RRT* algorithm, **(e)** MS-Bi RRT* algorithm, and **(f)** A*-MSRRT* algorithm.

In Environment B, the path trajectories generated by six distinct algorithms are presented in **Figures 5a–f**, with the trajectory obtained from the proposed method shown in **Figure 5f**. In comparison to other approaches, the proposed algorithm produces fewer redundant nodes, achieves a shorter flight path, and results in a smoother trajectory, thereby aligning more effectively with UAV flight demands. According to the data summarized in **Table 2**, the average trajectory generated by the proposed method results in a 56.3% decrease in computation time when compared to the MS-Bi RRT* algorithm. Furthermore, relative to the Bi-Informed-RRT* approach, it reduces the path length by 2.5% and the number of nodes by 25.3%, highlighting its enhanced search efficiency in more complex environments.

**Table 2:** Comparison of algorithm results in environment B.

Algorithm	Average path length	Average time	Average nodes
RRT*	12.18	20.99	4017
Bidirectional RRT*	12.71	15.03	3868
Bi-Informed-RRT*	11.93	40.27	3694
PBi-RRT*	11.74	25.87	3471
MS-Bi RRT*	11.87	8.01	3277
A*-MSRRT*	11.63	3.5	2761

The asterisk suffix in algorithm names (e.g., RRT, Informed-RRT, A-MSRRT) denotes the asymptotically optimal variant of the base algorithm. This notation originates from the Rapidly-exploring Random Tree Star (RRT)* algorithm proposed by Karaman and Frazzoli (2011), where the asterisk symbolizes its ability to converge to an optimal solution through iterative refinement. In motion planning contexts, the * suffix universally indicates that the algorithm guarantees asymptotic optimality.

**Figure 6:**
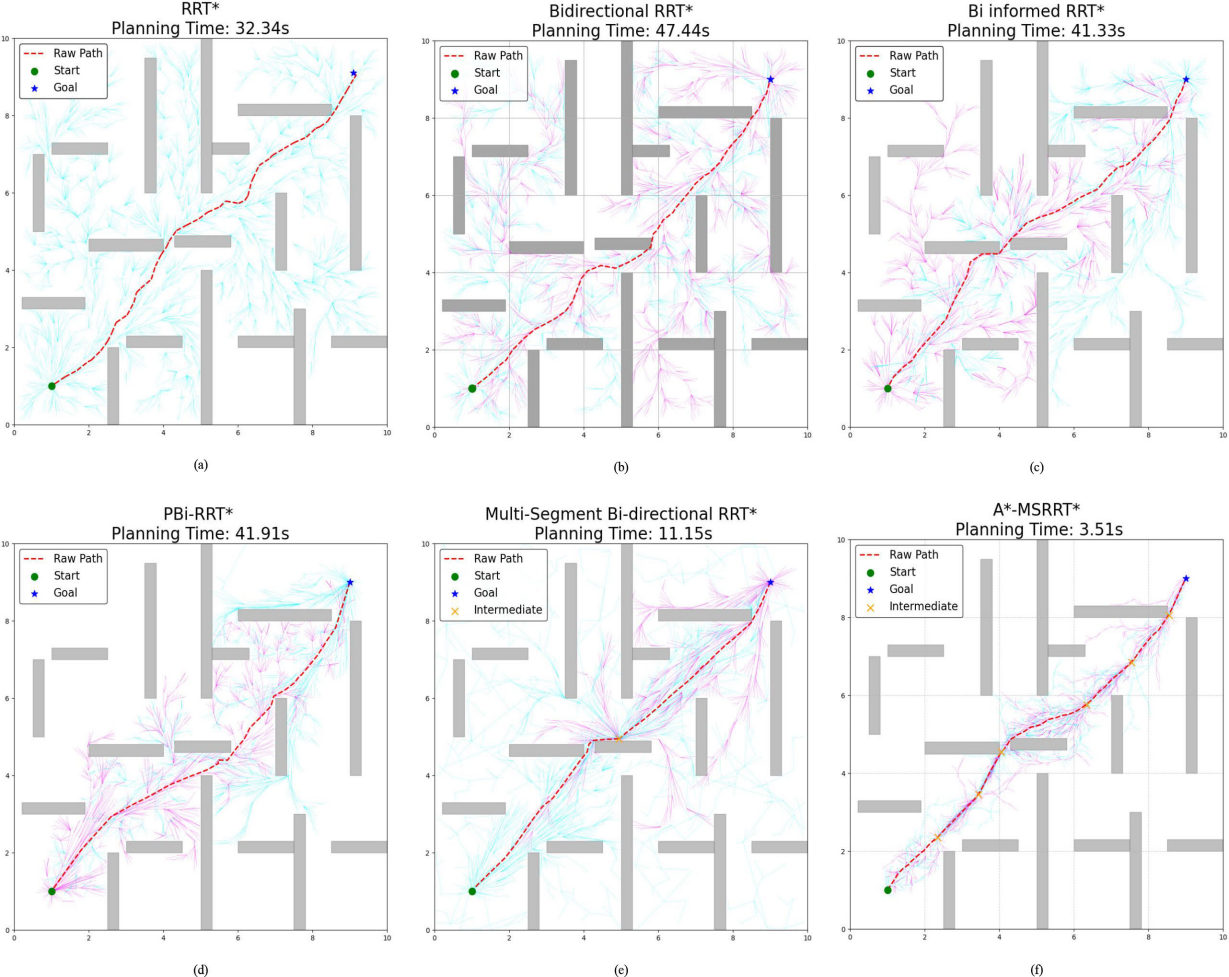
Environment C :high-density obstacles. **(a)** RRT* algorithm, **(b)** Bidirectional RRT* algorithm, **(c)** Bi-Informed-RRT* algorithm, **(d)** PBi-RRT* algorithm, **(e)** MS-Bi RRT* algorithm, and **(f)** A*-MSRRT* algorithm.

**Figure 7:**
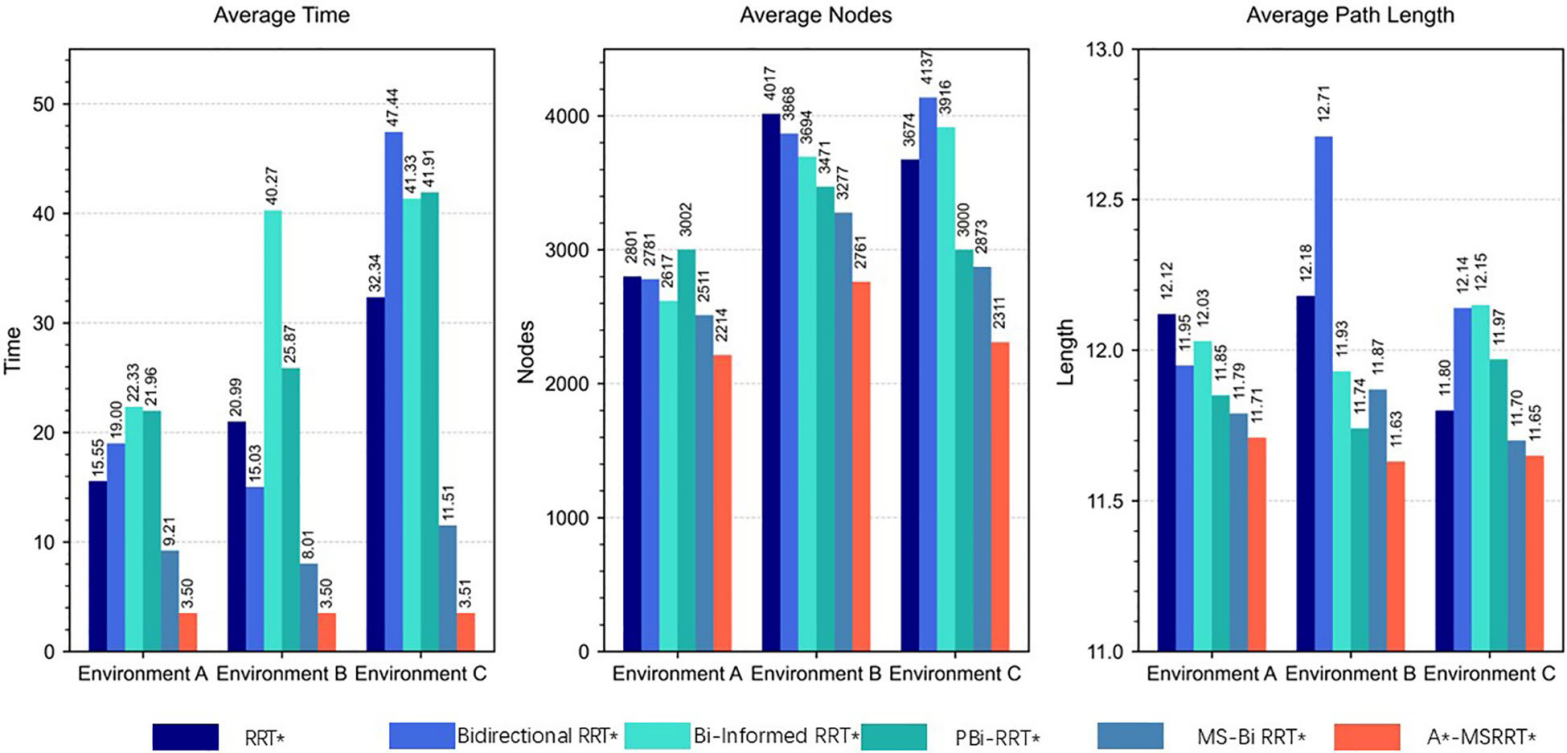
Comparison of result data.

In the more challenging obstacle-rich Environment C, the strengths of the proposed algorithm are more clearly demonstrated. While bidirectional search combined with elliptical sampling can reduce computation time, the generated paths still exhibit numerous sharp turns and remain less than optimal, as illustrated in **Figures 6a–e**. By contrast, the proposed method generates a significantly smoother trajectory under complex conditions, enables efficient bidirectional exploration, greatly shortens search time, and removes a considerable number of redundant nodes, as shown in **Figure 6f**. Based on the analysis of **Table 3** and **Figure 7**, the proposed algorithm achieves a 0.42% reduction in path length, a 68.5% decrease in computation time, and a 19.6% drop in the average number of nodes when compared to the MS-Bi RRT* algorithm. As a result, it effectively minimizes the generation of low-quality random samples and substantially improves computational efficiency.

**Table 3:** Comparison of algorithm results in environment C.

Algorithm	Average path length	Average time	Average nodes
RRT*	11.8	32.34	3674
Bidirectional RRT*	12.14	47.44	4137
Bi-Informed-RRT*	12.15	41.33	3916
PBi-RRT*	11.97	41.91	3000
MS-Bi RRT*	11.7	11.15	2873
A*-MSRRT*	11.65	3.51	2311

The asterisk suffix in algorithm names (e.g., RRT, Informed-RRT, A-MSRRT) denotes the asymptotically optimal variant of the base algorithm. This notation originates from the Rapidly-exploring Random Tree Star (RRT)* algorithm proposed by Karaman and Frazzoli (2011), where the asterisk symbolizes its ability to converge to an optimal solution through iterative refinement. In motion planning contexts, the * suffix universally indicates that the algorithm guarantees asymptotic optimality.

### 3.3 3D simulation

**Figure 8:**
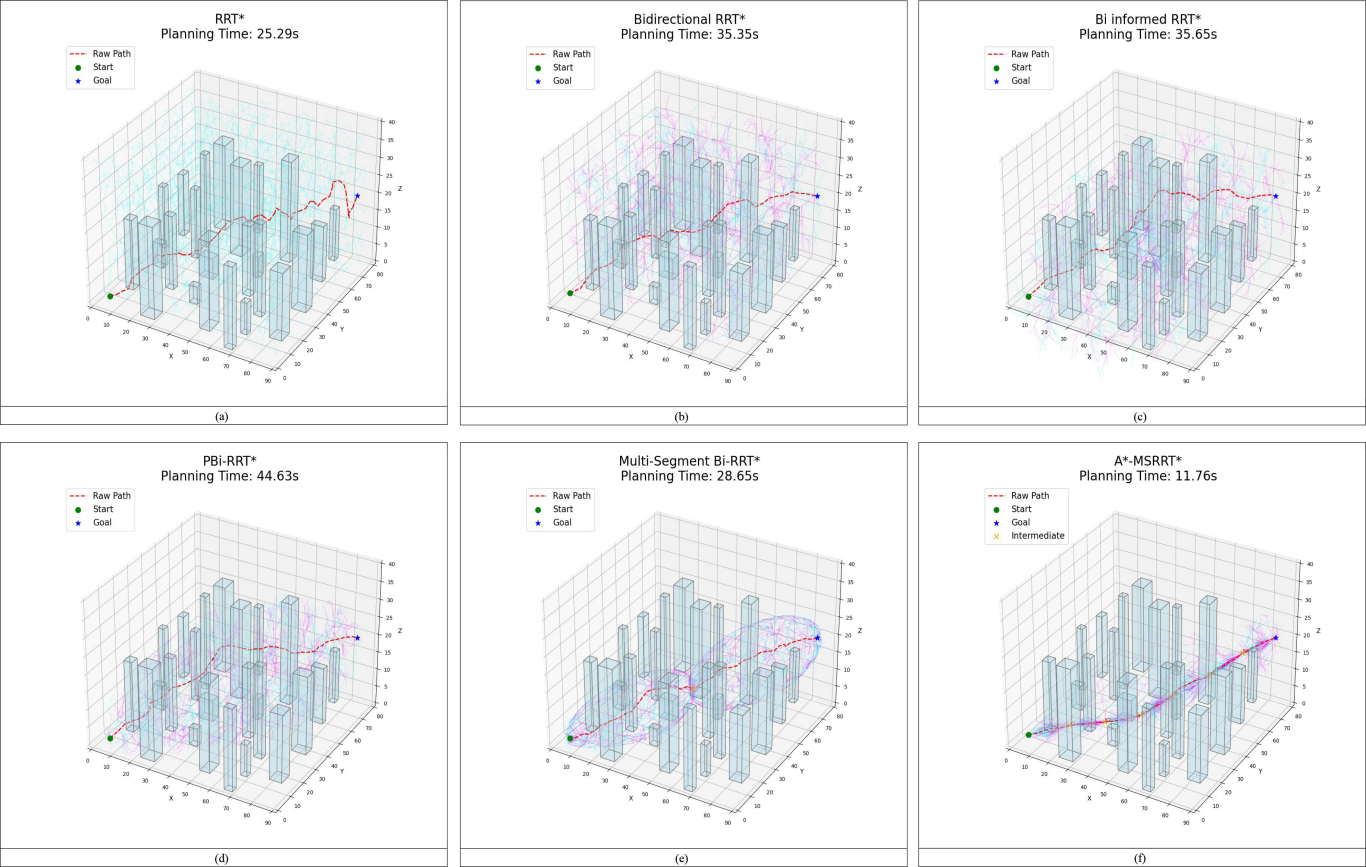
Path results in 3D environment. **(a)** RRT* algorithm, **(b)** Bidirectional RRT* algorithm, **(c)** Bi-Informed-RRT* algorithm, **(d)** PBi-RRT* algorithm, **(e)** MS-Bi RRT* algorithm, and **(f)** A*-MSRRT* algorithm.

To further demonstrate the proposed algorithm’s ability to reach the target quickly and stably in high-density obstacle and complex environments, the performance of six algorithms was compared in a 3D environment, as analyzed in **Figure 8** and **Table 4**. The RRT* algorithm exhibits faster computation time but requires a large number of nodes and produces longer paths, while the PBi-RRT* algorithm yields shorter paths but requires longer computation time. Compared with the relatively better-performing MS-Bi RRT* algorithm, the A*-MSRRT* algorithm reduces computation time by 58.9%, path length by 5.7%, and the number of nodes by 12.3%. The experimental results show that the A*-MSRRT* algorithm exhibits strong robustness under different obstacle densities. With respect to the three-core metrics—flight distance, computation time, and number of search nodes—the A*-MSRRT* algorithm exhibits consistent and stable performance, showing minimal sensitivity to variations in environmental conditions.

**Table 4:** Comparison of algorithm results in 3D environment.

Algorithm	Average path length	Average time	Average nodes
RRT*	149.11	25.29	6748
Bidirectional RRT*	135.39	35.35	5852
Bi-Informed-RRT*	128.89	35.65	5331
PBi-RRT*	118.19	44.63	5003
MS-Bi RRT*	115.54	28.65	4791
A*-MSRRT*	109.00	11.76	4202

The asterisk suffix in algorithm names (e.g., RRT, Informed-RRT, A-MSRRT) denotes the asymptotically optimal variant of the base algorithm. This notation originates from the Rapidly-exploring Random Tree Star (RRT)* algorithm proposed by Karaman and Frazzoli (2011), where the asterisk symbolizes its ability to converge to an optimal solution through iterative refinement. In motion planning contexts, the * suffix universally indicates that the algorithm guarantees asymptotic optimality.

## 4 Discussion

In the simulation setup, the environment is modeled on agricultural scenarios, where elongated obstacles represent fruit trees and other typical barriers found in such settings. To avoid collisions, flight paths are restricted from passing through these obstacle regions. The UAV trajectories planned using the A*-MSRRT* algorithm in Environments A, B, C, and the 3D setting are shown in **Figures 4f**, **5f**, **6f**, and **8f**, respectively. To evaluate its effectiveness, the A*-MSRRT* algorithm is compared against RRT*, Bidirectional RRT*, Bi-Informed-RRT*, PBi-RRT*, and MS-Bi RRT* algorithms. A*-MSRRT* yields lower fluctuations in results, particularly in high obstacle density scenarios, where both the path length and computation time remain within acceptable bounds, reflecting strong stability. Additional experiments on maps of varying dimensions further confirm the robustness of the A*-MSRRT* approach. While all algorithms demonstrate a general upward trend in average flight distance, processing time, and node count as obstacle density increases, A*-MSRRT* continuously outperforms others by maintaining a clear advantage in computational efficiency.

**Figure 9:**
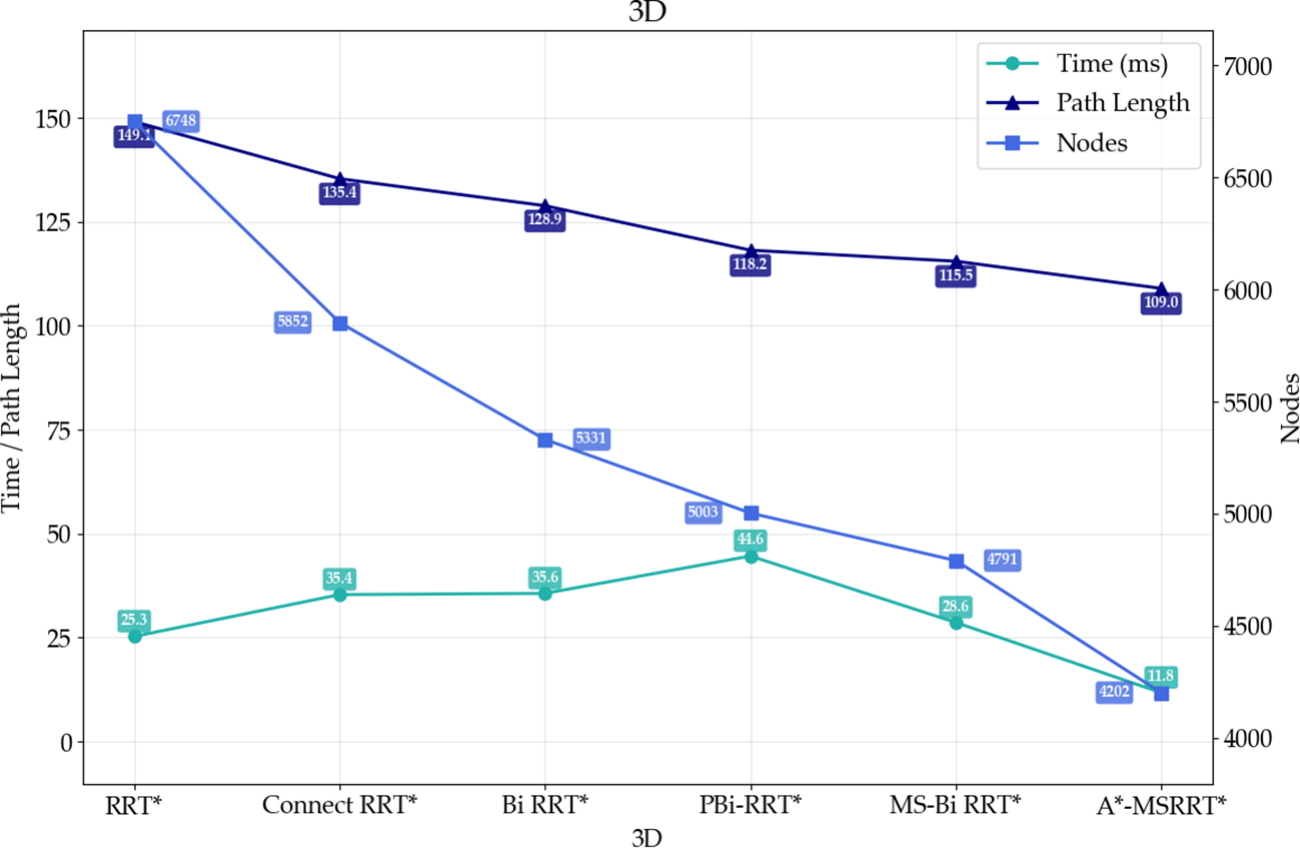
Comparison of path results in 3D environment.

Experimental results from **Figure 8** and data in **Figure 9** reveal that A*-MSRRT* achieves the shortest computation time, reducing it by approximately 53.5% to 73.6%, while also decreasing path length by 5.7% to 26.9% and lowering the number of search nodes by 12.3% to 37.7%. Overall, the A*-MSRRT* algorithm demonstrates a strong ability to balance path quality and computational efficiency, consistently yielding reliable and effective performance across diverse and complex environments.

The experiment first employs the A* algorithm to generate a coarse global path, from which key turning points are extracted as anchor nodes for segmenting the path. However, when applying the multi-segment bidirectional Informed-RRT* algorithm based on these key points, the resulting path exhibits pronounced angular features due to the lack of smooth transitions at segment junctions, thereby reducing path continuity and traversal efficiency. To address this issue, the study introduces a B-spline interpolation method for path optimization, utilizing a cubic interpolation function. The optimized trajectory is illustrated in Appendix S1 (**Supplementary Figure 5**), and the interpolation function is defined in Equation 32.


(32) $$\begin{array}{c} C(t)=\sum_{i=0}^{n-1}N_{i,k}(t)P_{i} \end{array}$$

Note: k denotes the order of the B-spline function, which is set to 3 in this study.、

To quantitatively evaluate the optimization effect of the path smoothing strategy, this study compares the original A*-MSRRT* algorithm with the improved approach incorporating B-spline interpolation sampling, as illustrated in Appendix S1 (**Supplementary Figure 6**). As shown in Appendix S1 (**Supplementary Table 5**), the B-spline method demonstrates a slight yet consistent reduction in path length across all tested environments. Specifically, in Environment A, the average path length is reduced from 11.811 to 11.797, corresponding to a relative improvement of approximately 0.12%. Similar improvements are observed in Environment B (from 11.577 to 11.571, 0.052%) and Environment C (from 11.60 to 11.59, 0.086%). These results indicate that the B-spline-based method effectively enhances path continuity and execution efficiency without altering the global path structure.

## 5 Conclusions

This paper presents an enhanced Informed-RRT* algorithm designed to tackle the challenge of high obstacle density commonly encountered by UAVs during plant phenotypic data collection. The proposed A*-MSRRT* algorithm improves upon the RRT* framework by introducing an intermediate node mechanism, enabling efficient and stable path planning in densely obstructed environments. During the path search process, obstacles are categorized into three types, and the algorithm integrates the A* strategy, a dynamic allocation mechanism for key intermediate nodes, and elliptical sampling constraints to guide UAVs safely toward their destinations. This improved method effectively mitigates the drawbacks of the original RRT* algorithm, such as excessive redundant points, high iteration counts, and unnecessarily long paths, thereby improving search performance. Simulation results demonstrate that the proposed A*-MSRRT* heuristic fusion approach achieves a minimum reduction of 0.6% in path length, 56.3% in computation time, and 11.8% in node count. Consequently, the algorithm better aligns with UAV flight requirements and outperforms the other five algorithms evaluated, indicating strong application potential. Nevertheless, the current implementation is limited to static 2D and 3D environments and single-UAV path planning. In real-world scenarios, the increasing prevalence of multi-UAV coordination in dynamic environments presents a promising direction for future research. In conclusion, this study provides not only robust technical support for UAV path planning in smart agriculture but also a solid theoretical foundation for the scalable application of heuristic-sampling-based algorithms in complex agricultural environments. By integrating deterministic search heuristics, dynamic node allocation strategies, and geometric constraints into the sampling-based planning framework, the proposed method demonstrates strong adaptability to environments with high obstacle density and spatial complexity. These properties suggest that the algorithm holds considerable potential for extension to multi-agent coordination, real-time replanning, and deployment in diverse precision agriculture scenarios, thereby advancing the practical adoption of UAV systems in real-world operations.

## Data Availability

The raw data supporting the conclusions of this article will be made available by the authors, without undue reservation.
