# Supplementary material for: Multi-stage bidirectional informed-RRT * plant protection UAV path planning method based on A * algorithm domain guidance
Source: Front Plant Sci. 2025 Aug 22;16:1650007. doi: 10.3389/fpls.2025.1650007 (PMC12411544; doi:10.3389/fpls.2025.1650007)
Supplement: Supplementary file 1 [file DataSheet1.pdf]

Supplementary Material

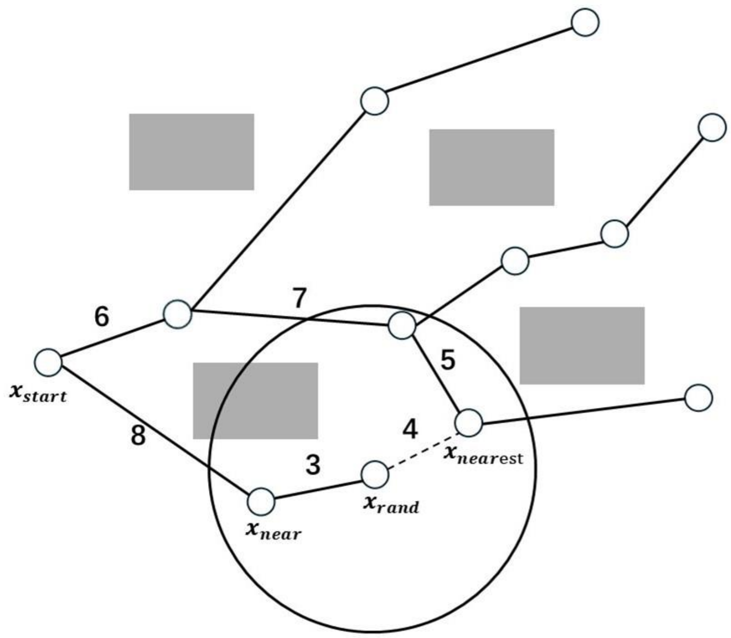

Supplementary Figure 1. The process of resetting parent nodes and rerouting in the RRT*algorithm.

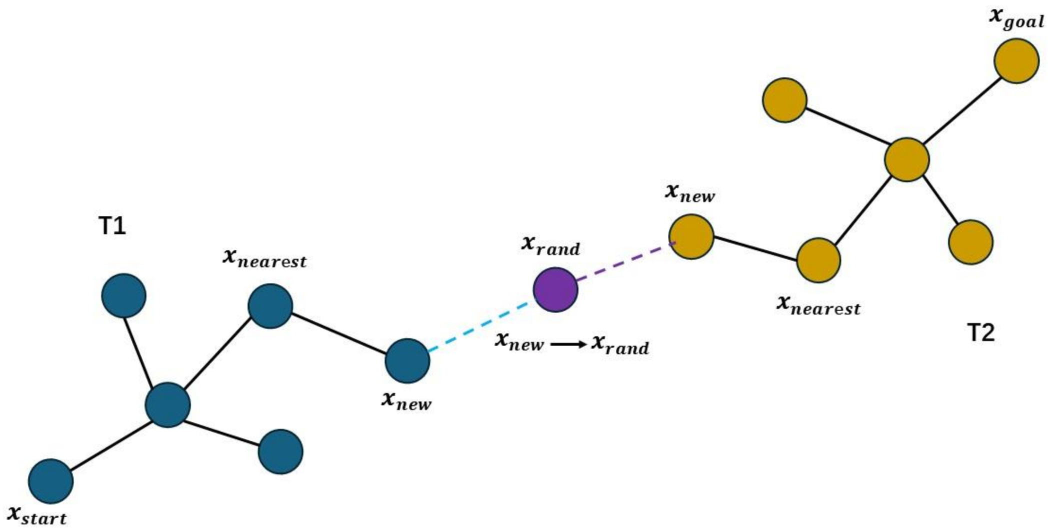

Supplementary Figure 2. The procedure for connecting trees in the bidirectional RRT* algorithm.

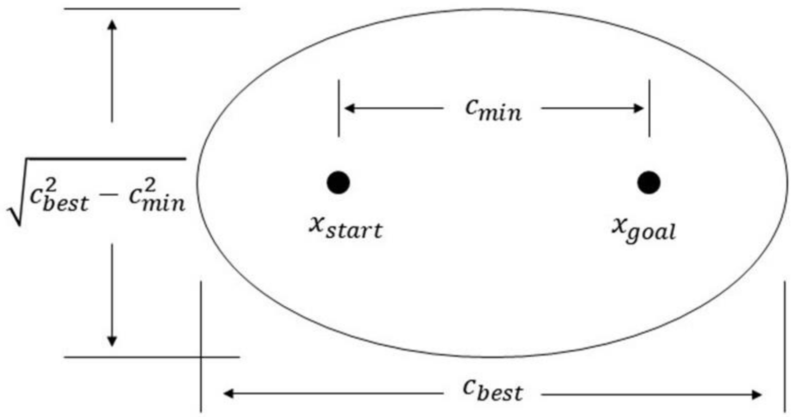

Supplementary Figure 3. Elliptical sampling

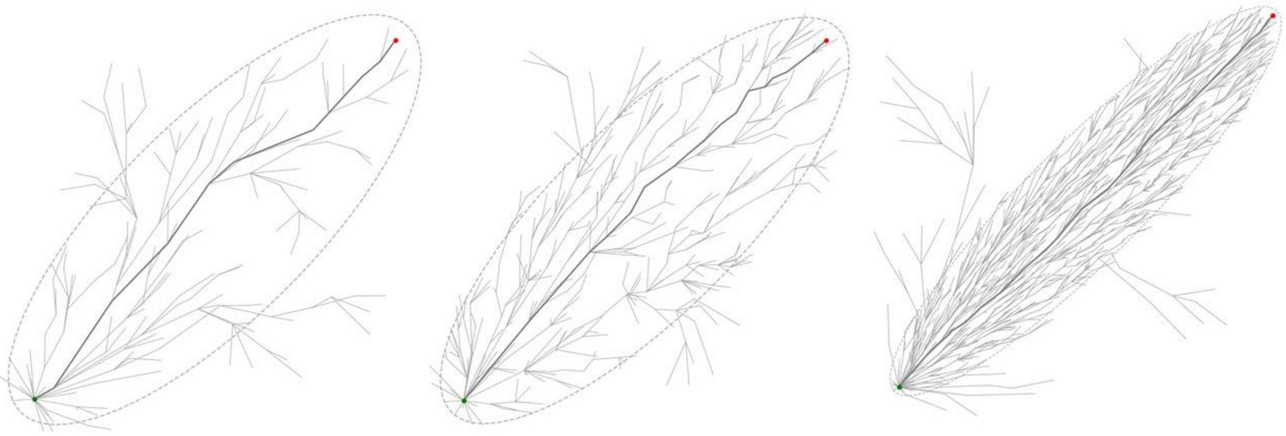

Supplementary Figure 4. Elliptical convergence process

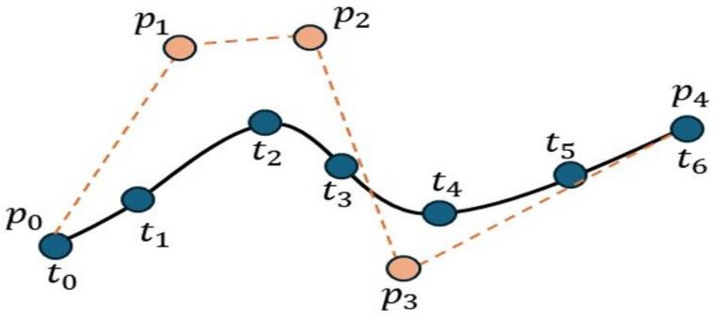

Supplementary Figure 5. B-spline sampling method

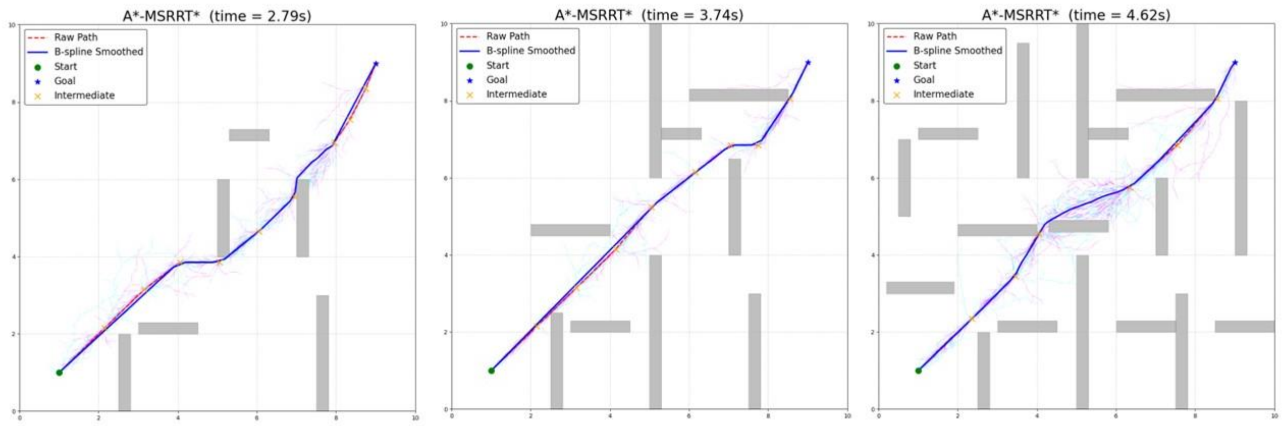

Supplementary Figure 6. Comparison of the optimized paths

Table 5: Comparison of optimized path data

Algorithm	Environment A	Environment B	Environment C
A*-MSRRT*	11.811	11.577	11.600
B-spline	11.797	11.571	11.590
